# Diverse metabolic reactions activated during 58-hr fasting are revealed by non-targeted metabolomic analysis of human blood

**DOI:** 10.1038/s41598-018-36674-9

**Published:** 2019-01-29

**Authors:** Takayuki Teruya, Romanas Chaleckis, Junko Takada, Mitsuhiro Yanagida, Hiroshi Kondoh

**Affiliations:** 1 G0 Cell Unit, Okinawa Institute of Science and Technology Graduate University (OIST), Okinawa, Japan; 2 Geriatric unit, Department of Community Network and Collaborative Medicine, Graduate School of Medicine, Kyoto University, Kyoto, Japan; 3 Present Address: Gunma University Initiative for Advanced Research (GIAR), Gunma University, Gunma, Japan

## Abstract

During human fasting, metabolic markers, including butyrates, carnitines, and branched-chain amino acids, are upregulated for energy substitution through gluconeogenesis and use of stored lipids. We performed non-targeted, accurate semiquantitative metabolomic analysis of human whole blood, plasma, and red blood cells during 34–58 hr fasting of four volunteers. During this period, 44 of ~130 metabolites increased 1.5~60-fold. Consistently fourteen were previously reported. However, we identified another 30 elevated metabolites, implicating hitherto unrecognized metabolic mechanisms induced by fasting. Metabolites in pentose phosphate pathway are abundant, probably due to demand for antioxidants, NADPH, gluconeogenesis and anabolic metabolism. Global increases of TCA cycle-related compounds reflect enhanced mitochondrial activity in tissues during fasting. Enhanced purine/pyrimidine metabolites support RNA/protein synthesis and transcriptional reprogramming, which is promoted also by some fasting-related metabolites, possibly via epigenetic modulations. Thus diverse, pronounced metabolite increases result from greatly activated catabolism and anabolism stimulated by fasting. Anti-oxidation may be a principal response to fasting.

## Introduction

Metabolic profiles of human blood provide valuable information about *in vivo* physiological states, which are influenced by genetic, epigenetic, physiological, and life-style factors^1–3^. Metabolomics, which detects, identifies, and quantifies small organic metabolites, is one of the rapidly developing domains of chemical biology, and constitutes a powerful tool in the search for useful diagnostic or bio-markers^4^. It permits comprehensive evaluation of metabolic mechanisms of physiological responses and diseases^5^ and of biological effects of drugs, nutrients, and environmental stressors^1^.

We previously established quantitative procedures to analyze metabolites of human whole blood, plasma, and RBCs (red blood cells) by LC-MS (liquid chromatography-mass spectrometry)^6,7^ based on our experience in developing metabolomic methods for fission yeast cells under various nutritional and genetic pertubations^8–11^. The software package, MZmine, is widely (~700 citations) used for non-targeted metabolomic analysis of both human and fission yeast samples^12^. Because non-targeted, comprehensive data have been very scarce in the literature (particularly for RBCs)^6^, we chose that approach to metabolites in fission yeast and blood. By comparing metabolomic profiles between young and elderly persons, we were able to identify age-related metabolites in both plasma and RBCs^7^.

Fasting is one of the most significant physiological stimuli to the human body, as nutrient limitation greatly affects energy production, triggering a wide range of catabolic reactions. The body’s glycogen storage capacity is limited and rapidly exhausted, and nutrients such as lipids are consumed as energy substitutes for glucose, which under non-fasting conditions, is employed as the major fuel source. After glycogen stores are depleted, gluconeogenesis is employed to maintain blood sugar levels. Radioisotope experiments have shown that constitutively activated gluconeogenesis accounts for the majority of glucose production in human body after prolonged fasting^13,14^. In addition to gluconeogenesis, evidence from serum or plasma suggests that fasting stress forces the human body to utilize various non-glucose metabolites, such as conversion of 3-hydroxybutyrate (3-HB) into acetyl-CoA, as energy sources^15,16^.

We analyzed metabolites during fasting, to monitor their changes. As most metabolic studies of fasting have tracked specific plasma or serum metabolites, such as butyrates, acylcarnitines, and branched-chain amino acids (BCAAs), our exhaustive, non-targeted analysis was intended to identify new fasting marker metabolites. Here we report non-targeted LC-MS analysis of whole blood, plasma, and RBCs during 58 hr of fasting. We found more than 30 previously unreported metabolites that change abundance significantly during fasting.

## Results

### Quantification of blood metabolites from 4 volunteers during fasting

**Figure 1:**
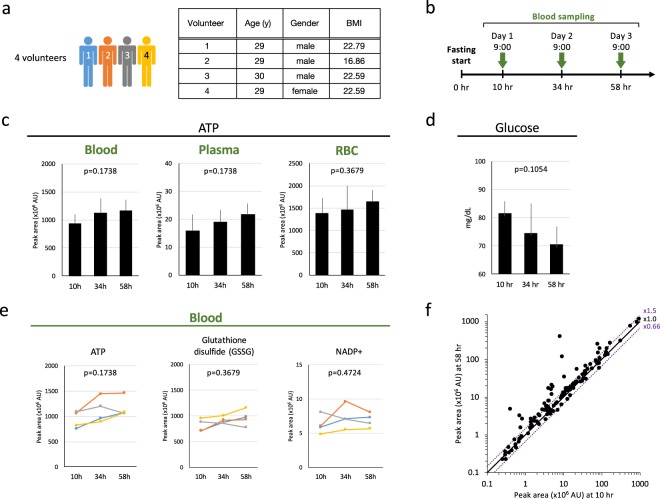
Quantification of blood metabolites from 4 volunteers during prolonged fasting. (**a**) Experimental procedures employed to study metabolomic changes during human fasting for 58 hr. Four, healthy young volunteers joined the study. The right panel shows age, gender, and BMI for each of four volunteers. (**b**) Blood samples from each person were taken at the indicated timepoints. Samples were immediately quenched in 50% methanol at −40 °C. Resulting extracts were used for metabolomic analysis. (**c**) Levels of ATP remained constant. (**d**) Blood glucose levels determined with a glucose tester during fasting. Whole blood glucose levels remained within the normal range (70–80 mg/dL) due to gluconeogenesis during fasting. (**e**) Levels of vital metabolites remained essentially constant during fasting. Profiles of ATP, glutathione disulfide (GSSG), and NADP^+^ in whole blood are shown. (**f**) Scatter plot of 120 metabolites between 10 and 58 hr of fasting. Average whole blood data are shown for the four volunteers. Compounds that displayed minor shifts (within 1.5x~0.66x) in abundance are placed between two purple lines. In each panel, p-values are presented to show the significance of serial change until 58 hr by Friedman test.

Blood samples were obtained from four young, healthy, non-obese volunteers. Obese people are not included in the present study, as obesity is known to affect the levels of some fasting markers, BCAAs and acylcarnitines^17^. Their ages, genders, and BMIs are shown in Fig. 1a. Phlebotomy was performed in the hospital at 10, 34, and 58 hr after fasting (Fig. 1b), to facilitate rapid preparation of metabolome samples. Immediately after blood collection, metabolome samples for whole blood, plasma, and RBCs were prepared separately, followed by metabolomic measurements by LC-MS^6^. Levels of ATP, an essential energy metabolite, did not change significantly in whole blood, plasma, or RBCs of the four volunteers throughout the fast (Fig. 1c). Plasma ATP levels were much lower than in RBCs or whole blood. All participants remained healthy and manifested no adverse symptoms during the study. Blood glucose levels of participants remained within the normal range (70–80 mg/dl) (Fig. 1d).

Comprehensive, quantitative analyses of blood metabolites were performed. Previously we identified 126 metabolites in human whole blood, approximately half of which were enriched in RBCs^6,7^. During 58 hr of fasting, the majority (62%) of these compounds were maintained at roughly constant levels. For example, levels of essential compounds, such as gluthathione, and NADP^+^ remained roughly constant, as in the case of ATP (Fig. 1e). The abundance of metabolites in three components (blood, plasma, and RBCs) are summarized in Table S1. Among 120 metabolites, five RBCs enriched metabolites (carnosine, NADP^+^, opthalmic acid, S-methyl-ergothioneine, trimethyl-tyrosine) were not detected in plasma, due to their low abundance in plasma.

### Increase of 44 blood metabolites and decrease of 2 during fasting

We employed non-targeted, comprehensive analysis of whole blood, plasma, and RBC metabolites using the software, MZmine 2^18^ for metabolite identification.

Overall metabolite changes between 10 and 58 hr are seen in a scatter plot (Fig. 1f), in which metabolite abundances that were significantly affected are shown as dots displaced from the diagonal line. Levels of ~37% of detected metabolites increased significantly (>1.5x). Table 1 lists 46 compounds that displayed statistically significant (<0.66x or >1.5x) shifts in abundance during 58 hr of fasting, whose calculated formal powers were shown in Table S2: among 46 metabolites, the statistical power for 38 compounds was found to be rather high (more than 0.8). In addition, the effect size *f* of Cohen’s logic^19^ obtained (Table S2) was large for the majority of metabolites. Among them, 32 metabolites have not previously been reported as fasting markers. Non-targeted analysis thus enabled us to find many new candidate fasting markers.

**Table 1:** Most metabolites changed peak areas significantly during fasting.

Category	Compounds	Blood			Plasma			RBC		
		peak area 10 h	fold-change		peak area 10 h	fold-change		peak area 10 h	fold-change	
			34 h/10 h	58 h/10 h		34 h/10 h	58 h/10 h		34 h/10 h	58 h/10 h
Butyrates (4)	2-Hydroxybutyrate	L	7.1	13.8	M	6.7	13.7	L	6.0	11.1
	*2-Ketobutyrate*	L	6.7	12.7	L	4.9	11.7	L	4.8	8.1
	3-Hydroxybutyrate	L	19.9	58.0	L	17.2	55.4	L	16.3	44.9
	Aminobutyrate*	L	1.8	5.0	L	3.7	4.7	L	3.1	6.1
BCAAs (5)	Isoleucine	M	1.6	2.1	M	1.4	1.8	M	1.3	1.7
	Keto(iso)leucine	M	2.5	3.4	M	2.3	3.7	M	2.4	3.1
	*Ketovaline*	M	2.6	3.4	M	2.4	3.7	L	2.4	2.9
	Leucine	M	2.0	2.3	M	1.7	2.0	H	1.5	1.9
	Valine	M	1.7	2.0	M	1.4	1.9	M	1.3	1.6
Acylcarnitines (7)	Acetyl-carnitine*	L	1.6	2.1	L	2.9	5.3	L	1.1	1.4
	Decanoyl-carnitine	M	6.7	4.8	M	6.3	7.2	L	5.8	7.0
	Dodecanoyl-carnitine	L	5.1	4.7	L	5.8	8.1	L	3.7	5.5
	*Hexanoyl-carnitine*	L	2.2	2.0	L	3.0	3.8	L	1.6	1.9
	*Isovaleryl-carnitine*	M	1.2	1.2	L	1.3	1.8	M	1.2	1.4
	Octanoyl-carnitine	L	8.0	6.1	L	8.0	8.8	L	5.4	6.3
	Tetradecanoyl-carnitine*	L	7.0	6.7	L	8.6	14.0	L	4.0	6.1
Organic acids (5)	*2-Oxoglutarate*	L	1.7	2.3	L	1.5	1.7	L	6.2	8.4
	*cis-Aconitate*	L	1.5	1.7	L	1.6	1.8	L	1.6	1.7
	Citrate	M	1.5	1.6	M	1.5	1.5	M	1.4	1.5
	Malate*	M	1.5	1.6	L	1.7	1.9	M	1.3	1.4
	Succinate*	L	1.5	1.5	L	1.5	1.3	L	1.4	1.5
Coenzymes (2)	Nicotinamide*	L	1.7	1.4	L	1.6	1.7	L	1.5	1.1
	Pantothenate*	L	1.5	1.7	L	1.8	2.6	L	1.2	1.4
Purines/Pyrimidines metabolism (9)	Adenine*	L	2.0	3.2	L	1.2	1.1	L	2.3	1.5
	ADP*	H	1.4	1.5	L	1.3	1.5	H	1.1	1.3
	*CTP*	L	1.1	1.1	L	1.4	1.6	L	1.2	1.0
	*Cytidine*	L	1.7	1.5	L	1.3	1.1	L	1.5	1.7
	GTP*	M	1.3	1.3	L	1.7	2.4	M	1.1	1.2
	IMP*	L	1.3	1.2	L	1.4	2.1	L	1.0	1.0
	Urate*	H	1.5	1.7	H	1.2	1.6	H	1.3	1.6
	Uridine	L	2.6	2.8	L	2.3	2.6	L	2.2	2.4
	*Xanthine*	L	3.5	4.0	L	3.8	4.4	L	2.8	3.5
Sugar metabolites (7)	6-Phosphogluconate*	L	1.4	1.5	L	1.5	1.7	L	1.0	1.2
	Diphosphoglycerate*	H	1.4	1.4	L	1.2	1.8	H	1.1	1.2
	Gluconate* ↓	M	1.2	1.2	L	0.8	0.6	M	1.0	0.8
	Glucose-6-phosphate*	M	1.2	1.3	L	1.5	1.6	M	1.0	1.2
	Glycerol-phosphate*	L	1.7	1.7	L	1.5	1.5	L	1.2	1.2
	Pentose-phosphate*	L	1.5	1.8	L	1.6	1.9	L	1.0	1.3
	Sedoheptulose-7-phosphate*	L	1.1	1.2	L	1.8	2.2	L	0.9	1.1
Anti-oxidants (3)	Ergothioneine*	M	1.2	1.2	L	1.5	1.9	H	1.1	1.2
	Ophthalmic acid*	L	2.0	3.6	not detected			L	1.8	3.2
	Carnosine*	L	1.1	0.8	not detected			L	1.3	1.8
Amino acids (4)	Aspartate* ↓	M	1.2	1.2	L	0.5	0.4	M	1.0	1.2
	*Dimethyl-arginine*	M	1.4	1.3	M	1.3	1.4	M	1.4	1.7
	*Lysine*	M	1.5	1.3	M	1.5	1.2	M	1.2	1.0
	*N-Acetyl-(iso)leucine*	L	1.3	1.0	L	1.3	1.1	L	1.2	1.7

Summary of 46 metabolites in whole blood, plasma, and RBCs that changed concentration significantly (>1.5x) during 58 hr of fasting. Thirty-two metabolites in *italics* have not been previously reported as fasting markers. Asterisks indicate 22 RBC-enriched metabolites. Note that some RBC-enriched compounds are also detectable in plasma^6^. Peak abundance, defined as high (H) (>10^8^ AU), medium (M) (10^7^–10^8^ AU), or low (L) (<10^7^ AU). Metabolites are classified as butyrates, acylcarnitines, BCAAs, amino acids, purines and pyrimidines, coenzymes, organic acids, antioxidants, and sugar-metabolites. Changes of abundance for each compound are indicated. Most metabolites increased during fasting, while only two decreased <0.66x (aspartate and gluconate), as indicated by arrows ↓.

The abundance of blood metabolites at 10 hr was categorized as H (high), M (medium), or L (low)^6^. None of the metabolites listed was comparable in abundance to ATP (H) in RBCs. All of the 46 compounds increased, except for aspartate (0.4-fold decrease) and gluconate (0.6-fold decrease) (Fig. S1a). All four volunteers showed similar patterns of decrease.

### Fasting-induced increase of four butyrates

**Figure 2:**
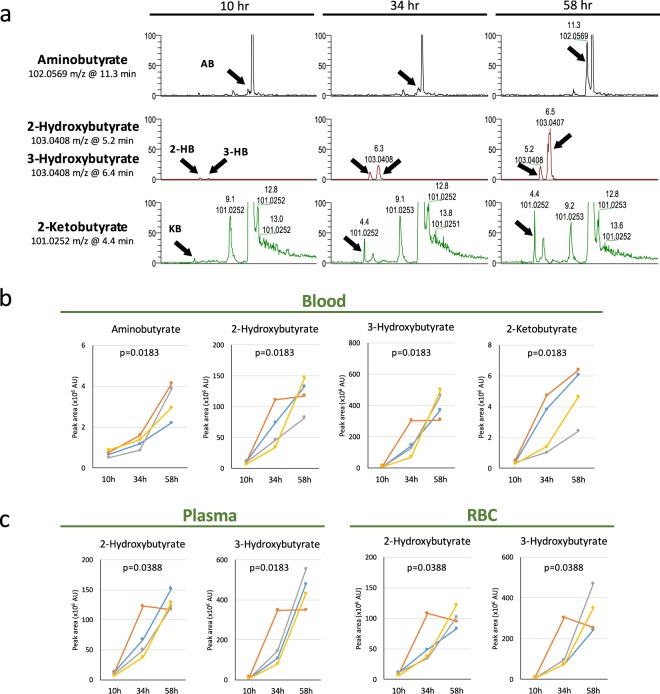
Four butyrates increased significantly during 58 hr of fasting. (**a**) Titers of four butyrates (indicated by arrows) increased strikingly during fasting. Importantly, these compounds were negligible before fasting and were not listed in our previous non-fasting study^7^. Peak areas increased sharply during fasting, becoming major peaks after 58 hr. Identification of compounds was verified using standards with MS/MS^6^. (**b**) Peak area changes of the four butyrates in whole blood samples of four volunteers. (**c**) Increases of 2- and 3-hydroxybutyrate in plasma and RBCs during fasting. In each panel, p-values are presented to show the significance of serial change until 58 hr by Friedman test.

We found four compound peaks that were nearly invisible 10 hr after fasting, but which later increased greatly, two of them becoming major peaks after 34 and 58 hr of fasting (Fig. 2a). Identifying these peaks using standards, the four were identified as aminobutyrate, 2- and 3-hydroxybutyrate (2-HB and 3-HB), and 2-ketobutyrate (KB). The first three have previously been reported as fasting markers^16,20^, but KB is novel. In plasma, KB increased 4.9- and 11.7-fold at 34 and 58 hr, respectively. In Rubio *et al*.^20^, 2-HB and 3-HB increased in concentration, respectively, from 0.03 mM (12 hr) to 0.16 mM (36 hr after fasting) and from 0.07 mM (12 hr) to 1.17 mM (36 hr after fasting). Our measurements (Table 1) closely corroborate those of Rubio *et al*. 2-aminobutyrate (2-AB) increased 2-fold in the study of Rubio *et al*., while our measurement was 3.7-fold. Note that after 58 hr of fasting, the average levels of 2-HB and 3-HB in four volunteers showed further increases (13.7- and 55.4-fold for 2-HB and 3-HB, respectively), indicating that blood concentrations of 2- and 3-HB after prolonged fasting reach exceedingly high concentrations (~5 mM 3-HB).

The striking increase of these four butyrates was observed in all 4 individuals (Fig. 2b,c), although absolute abundances varied among them. Curiously, the further increase of 2-HB and 3-HB from 34 to 58 hr did not occur in volunteer 2 (see Discussion section).

### Fasting-induced increases of BCAAs and carnitines

**Figure 3:**
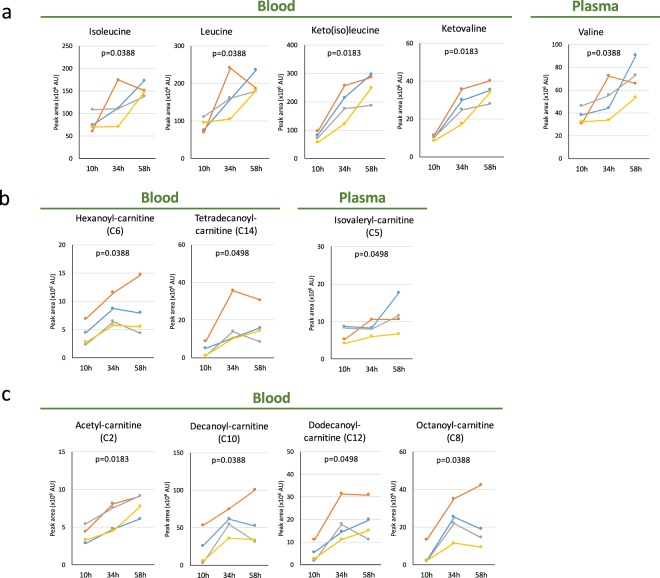
Profiles of BCAAs and carnitines abundant during fasting. (**a**) Profiles of BCAAs in blood during fasting. Ketovaline is identified as a novel fasting marker, although increases of isoleucine, leucine, valine, and keto(iso)leucine were reported previously. (**b**) Profiles of four carnitines. Left-hand panels display profiles for hexanoyl- and tetradecanoyl-carnitine (C6 and C14, respectively) in whole blood, while righthand panels show isovaleryl-carnitine (C5) in plasma. (**c**) Profiles of four other carnitines. Acetyl-, decanoyl-, dodecanoyl-, and octanoyl-carnitine have previously been identified as fasting markers. In each panel, p-values are presented to show the significance of serial change until 58 hr by Friedman test.

Branched-chain amino acids (BCAAs) are known as fasting markers^21–23^. Because BCAAs are converted to CoA compounds and used for energy generation via the Krebs cycle, they are implicated in mitochondrial activation. In our analysis, we found a novel BCAA fasting marker, ketovaline, in addition to isoleucine, keto(iso)leucine, leucine, and valine, which were previously known (Figs 3a and S2). These compounds are detected in blood before fasting and the degree of increase after 58 hr of fasting was moderate (2.0–3.4-fold increase; Table 1). After 58 hr fasting, ketovaline and ketoisoleucine increased the most (average 3.4-fold, Table 1) in all four volunteers (Fig. S3).

**Figure 4:**
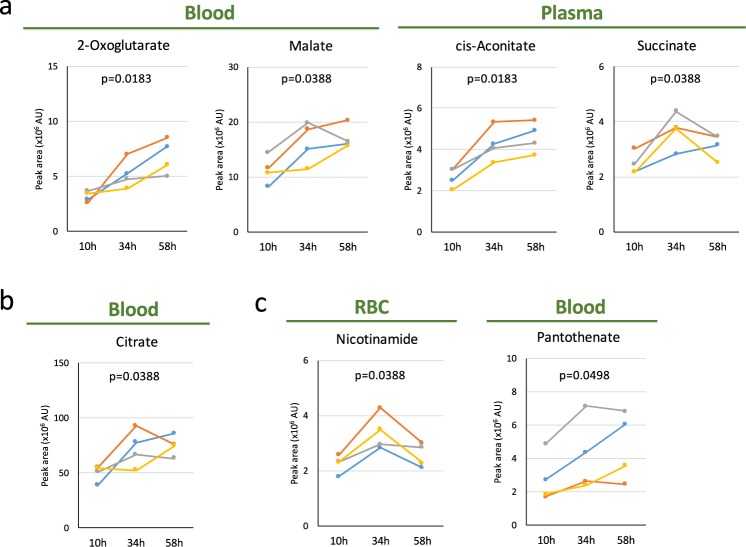
Profiles of organic acids and vitamins during 58 hr of fasting. (**a**,**b**) Increased levels of organic acids during fasting. 2-oxoglutarate, malate, cis-aconitate, and succinate are newly identified as fasting markers (**a**) in addition to confirmation of a previously reported increase of citrate (**b**). (**c**) Increased levels of nicotinamide in RBCs and pantothenate (a precursor for CoA) in whole blood. In each panel, p-values are presented to show the significance of serial change until 58 hr by Friedman test.

We also found that hexanoyl-, isovaleryl- and tetradecanoyl-carnitines may be novel fasting markers (Fig. 3b). Acylcarnitines are also major fasting metabolites, as are butyrates (Figs 3b,c, S2b,c and Table 1)^20,23,24^. Acylcarnitines function as lipid carriers, supporting lipid metabolism and reflecting β-oxidation activity in mitochondria^25,26^. Even though all four volunteers were young and healthy, the degree of increase (1.6~14-fold) reflected individual variability in carnitine functions in blood and tissues. The 15 metabolites displaying the most significant changes are listed in Fig. S3, in order of magnitude of change. This list largely contains butyrate derivatives, acylcarnitines, and BCAAs, consistent with previous work. However, volunteers 1, 3, and 4 displayed more prominent changes in butyrates and acylcarnitines than in BCAAs, while volunteer 2 showed greater increases in BCAAs than in acylcarnitines. Because volunteer 2 had Body Mass Index (BMI) below the lower limit (18.5) of the normal range (only 16.86), his lipid stores may not have been sufficient, so that his supplies of 2- and 3-HB were also lower than normal (Fig. 2c). In addition, the changes of GSSG (Fig. 1e), tetradecanoyl-carnitine (Fig. 3b), dodecanoyl-carnitine (Fig. 3c) and malate (Fig. 4a) from 34–58 hr in volunteer 3 were different from those in volunteer 1 and 4, by unknown reasons.

### Increase of organic acids and coenzymes

In the present study, we identified other classes of compounds that underwent significant changes, such as purines and pyrimidines, coenzymes, organic acids, anti-oxidants, and sugar metabolites (in plasma, not RBCs), revealing hitherto unrecognized aspects of fasting.

Roughly 2-fold increases in several organic acids (cis-aconitate, malate, 2-oxoglutarate, and succinate) were observed (Fig. 4a). These are involved in the TCA cycle. Taken together with the increase of citrate, which has been previously reported (Fig. 4b)^20^, mitochondrial activity in tissues may be activated during fasting. As human RBCs have neither mitochondria nor TCA cycle activity, these organic acids are likely to have been derived from tissues. Notably, coenzymes (nicotinamide and pantothenate, a precursor for acetyl-CoA) were also upregulated (Fig. 4c). Nicotinamide is essential for production of NADH and NADPH, while pantothenate serves as a precursor for production of Coenzyme A (CoA).

### Increases of pyrimidines and purines

**Figure 5:**
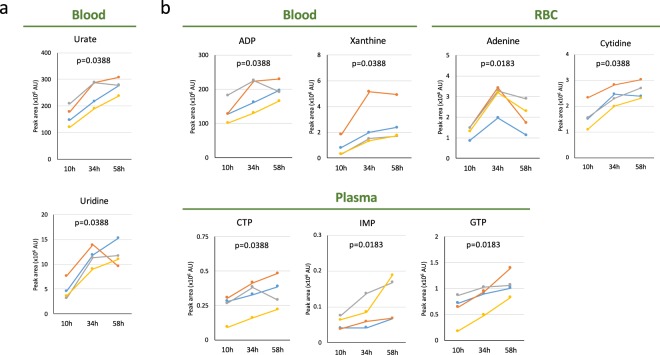
Profiles of pyrimidines and purines during 58 hr of fasting. (**a**) Increased concentrations of urate and uridine in blood were observed. (**b**) Upper panels show changes of ADP, xanthine (precursor for urate) in whole blood, and adenine, cytidine in RBCs, while lower panels indicate those of CTP, IMP, and GTP (in plasma). In each panel, p-values are presented to show the significance of serial change until 58 hr by Friedman test.

Urate and uridine are known to increase during fasting^2,27^, as we confirmed (Fig. 5a). Urate was the most abundant nucleoside or nucleotide detected during fasting (Table 1 and Fig. 5). All four volunteers clearly showed 1.5~1.7-fold increases in urate. In addition, GTP, CTP, ADP, IMP, cytidine, adenine, and xanthine (a precursor of urate) showed statistically significant increases (Fig. 5b). Increases of GTP, IMP, and CTP were found only in plasma (Fig. 5b). The 1.5-fold increase of ADP in blood was significant, judging from the peak area (~10^8^). Increases of GTP (2.4-fold in plasma), uridine, and xanthine (2.8- and 4.0-fold in blood, respectively) were also prominent (Table 1).

### Increased levels of pentose phosphate pathway (PPP) metabolites and antioxidants

**Figure 6:**
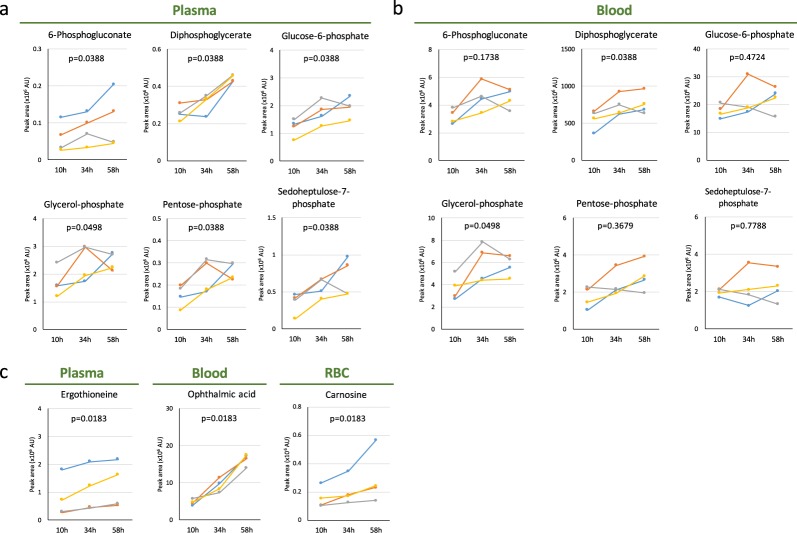
Profile of sugar phosphates and anti-oxidants during 58 hr of fasting. (**a**) Previously unreported changes in sugar phosphates were observed in plasma. 6-phosphogluconate, glucose-6-phosphate, pentose phosphate, and sedoheptulose-7-phosphate are intermediates in the pentose phosphate pathway. (**b**) Profile of sugar phosphates in whole blood. (**c**) Anti-oxidants increased during fasting: ergothioneine in plasma (left panel) and ophthalmic acid (OA) in whole blood (centre panel), and carnosine in RBCs (right panel). OA is produced by the same enzymes required to generate glutathione. Aminobutyrate, the precursor of OA, also increased (Fig. 2b) in parallel with upregulation of OA, while the level of glutathione disulfide (GSSG) remained constant (Fig. 1e). In each panel, p-values are presented to show the significance of serial change until 58 hr by Friedman test.

An additional new finding is that six sugar phosphates (6-phosphogluconate, diphosphoglycerate, glucose-6-phosphate, glycerol-phosphate, pentose-phosphate, phosphoglycerate and sedoheptulose-7-phosphate) increased more in *plasma* than in whole blood (Fig. 6a,b). Even small increases could be detected in plasma as their plasma titers were rather low under non-fasting conditions, whereas their levels in RBCs and consequently in whole blood were much higher. Significantly, of these, 6-phosphogluconate, glucose-6-phosphate, pentose-phosphate, and sedoheptulose-7-phosphate are generated in the pentose phosphate pathway (PPP), and are reported to be essential for redox maintenance and nucleic acid synthesis^28^.

Consistent with increases in PPP metabolites, the anti-oxidants, ergothioneine, and carnosine also greatly increased (Fig. 6c). Another interesting example is that of a tripeptide analog of glutathione, L-γ-glutamyl-L-α-aminobutyrylglycine, also known as ophthalmic acid (OA) (Fig. 6c). Synthesis of OA employs the same enzymes utilized for glutathione production^29,30^. Interestingly, the level of OA significantly increased, while that of glutathione remained constant (Fig. 1e). Although not previously reported, we also observed increases of lysine, dimethyl-arginine, and N-acetyl-(iso)leucine (Fig. S4). The physiological significance of these changes remains to be determined.

## Discussion

**Figure 7:**
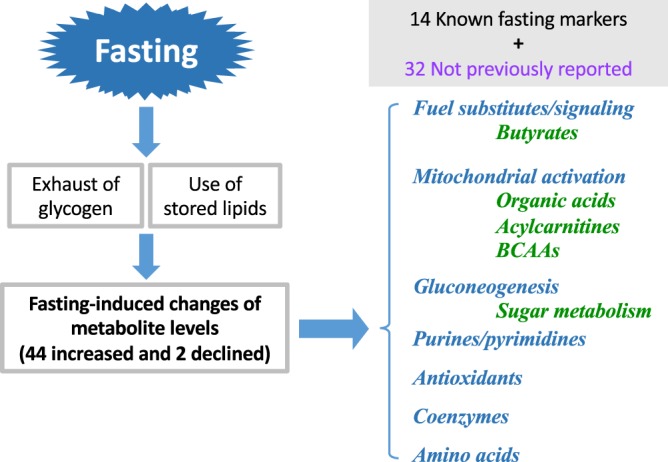
Summary of changes in the blood metabolome during 58 hr of fasting. Prolonged human fasting causes a much more metabolically active state than previously recognized. It has long been known that prolonged fasting exhausts glycogen stores (glucose), consumes stored lipids as fuel substitutes, and stimulates gluconeogenesis. Fourteen confirmed markers such as butyrates, acylcarnitines, and BCAAs increased. In this study, 32 new fasting marker metabolites, were identified. They are catabolized as butyrates, organic acids, sugar metabolites relevant to gluconeogenesis, acylcarnitines, BCAAs, amino acids, purines/pyrimidines, antioxidants, and coenzymes. These novel markers reveal possible new metabolic aspects of fasting. Increased levels of organic acids may reflect increased mitochondrial activity, from which ROS are counteracted by antioxidant production; ergothioneine (EG), ophthalmic acid (OA), pentose-phosphate-pathway (PPP) and others. Purine and pyrimidine anabolism is increased for RNA and protein synthesis. Moreover, not only 3-HB (a histone deacetylase inhibitor), but also purines, pyrimidines, and 2-oxyglutarate (2-OG) function may participate in signal reprogramming.

In this non-targeted, comprehensive analysis of human blood metabolites during prolonged fasting, we observed increases of a number of metabolites, such as organic acids, coenzymes, antioxidants, purines and pyrimidines that have rarely been linked to fasting. These appear to implicate hitherto unrecognized metabolic mechanisms induced by fasting: antioxidative defense (ergothioneine, OA, carnosine), enhanced mitochondrial activity (organic acids in the TCA cycle), increased purine and pyrimidine anabolism, pentose phosphate pathway (sedoheptulose-7-phosphatase, pentose phosphate), and remodeling of signaling (hydroxybutyrate). These metabolites are likely required for energy substitutes, such as for gluconeogenesis and TCA cycle intermediates, but also for synthetic pathways, such as for protein and nucleic acid synthesis. Thus, metabolic mechanisms during prolonged fasting reflect a well-balanced interplay between catabolic and anabolic reactions (Fig. 7). This non-targeted comprehensive analysis of human blood disclosed large-scale shifts in blood metabolite profiles during 58 hr of fasting, comprising significant changes in 46 metabolites, including 32 previously unreported fasting markers (Table 1). Only 14 compounds have previously been reported as fasting markers. Our results are consistent with previously reported changes in targeted-metabolite levels (butyrates, carnitines, BCAAs, citrate, uridine, and urate)^2,16,20,27^, which in turn, validate the protocol used here. Since the 44 metabolites account for one-third of all blood metabolites detected, fasting clearly caused major metabolic changes in human blood. Interestingly, non-targeted metabolomic analysis during mice fasting revealed overlapping but distinct profiles, compared to those in human fasting (our unpublished results). Significant increases in various classes of metabolites (butyrates, carnitines, BCAAs, organic acids) are possibly required not only as energy substitutes, but also for active gluconeogenesis, to maintain blood glucose levels during fasting^31^.

Due to the severe protocol of 3 day fasting, only four young healthy people joined the study as the pilot and exploratory one. We observed consistent and diverse individual variability in fasting response, which could be explained partly by their similarity or differences in BMI, lipid storage, and sex. For example, the earlier burst of butyrates and BCAAs for volunteer 2 could be due to low BMI than others. Although the comparison between 34 hr and 58 hr showed no differences in most metabolites except butyrates and OA, we also noticed that some metabolites peaked in 34 hr, while other metabolite kept increasing until 58 hr (cis-aconitate, succinate, nicotinamide (Fig. 4), adenine (Fig. 5), and lysine (Fig. S4). It is possible that unknown compensatory mechanism might operate during fasting to keep the levels of metabolites after 34 hr. Compared to larger studies, it is difficult to gain further mechanistic insight in our study, including individual variability of fasting response. Such limitation of our study should be resolved in the future.

First, increases of several antioxidants (OA, ergothioneine, carnosine, urate, and xanthine) revealed another aspect of metabolic remodeling during fasting. OA is an analog of glutathione, in which cysteine is replaced by 2-AB^30^. Consistently, aminobutyrate also significantly increased, while levels of glutathione during starvation remain rather constant. It may be that the level of glutathione is sufficiently vital to be tightly regulated even during fasting, while fasting boosts OA levels in parallel with the increase of 2-AB. Interestingly, increases of antioxidants (OA and ergothioneine) during fasting are evolutionarily conserved in both humans and fission yeast^8^. This could be partly due to increased oxidative stress under glucose-depleted conditions, since glycolysis also serves in anti-oxidative defense^32^. Thus, we speculate that increased OA reflects increased demand for antioxidants during fasting. Consistently, metabolites of the PPP, which is essential for redox maintenance via NADPH generation, increased in plasma, but not in RBCs. Our previous RBC metabolomics identified sugar phosphates and high-energy compounds as RBC-enriched; therefore, PPP metabolite increases only in plasma suggest that responses in tissues are largely responsible for these altered profiles during fasting. Increased sugar metabolites, which have scarcely been detected in previous metabolomic studies, may also reflect enhanced gluconeogenesis. These antioxidant metabolites are not covered in previous targeted studies on fasting. Collectively, these findings provide the first support for the notion that increased antioxidative defense is a significant physiological response during fasting. Next, some metabolites that changed significantly during fasting, suggest physiological impacts of fasting specifically on mitochondria, where fatty acids are metabolized by β-oxidation. Our identification of ketovaline (BCAA) and four acylcarnitines (isovaleryl-, hexanoyl-, tetradecanoyl-carnitine) as fasting markers, supports the notion that increased BCAAs and carnitines are required to maintain the metabolic activity of mitochondria through their conversion to acetyl-CoA and the transport of fatty acids into mitochondria, respectively. Interestingly, ketobutyrate (butyrates), identified here, have also been reported as metabolic intermediates leading to succinyl-CoA^33^. Moreover, we observed increases of several organic acids (cis-aconitate, citrate, malate, 2-oxoglutarate, and succinate). Thus, global increases of TCA cycle-related compounds reflect enhanced mitochondrial activity in liver, muscle, kidney, or other tissues^34^, since human RBCs lack mitochondria. As aspartate significantly decreases during fasting, malate-aspartate shuttle might also support TCA cycle activation. Alternatively, its conversion to phosphoenolpyruvate through oxaloacetate might be available for replenishing glucose via gluconeogenesis^35^. Third, increases of high-energy compounds (CTP, IMP, and GTP), purine/pyrimidine metabolites, in plasma only, imply that blood metabolomic changes during fasting reflect metabolic changes in various tissues, but apparently not in RBCs. As purine/pyrimidine metabolites are required for transcriptional reprogramming^36^, some anabolic metabolisms for RNA and protein synthesis would also be supported by increases of purines and pyrimidines. Moreover, increased purine/pyrimidine metabolism probably supports not only energy production, but also antioxidant synthesis^37^, and possibly neuroprotection^38,39^. Fourth, some fasting-related metabolites may function as signaling modules to maintain physiological homeostasis during fasting. We identified an increase of possible signaling modules (3-HB and 2-oxoglutarate) during fasting. 3-HB, a well-established fasting marker (Fig. 2 and Table 1), is also known as a histone deacetylase inhibitor^40^, as is the related sodium butyrate^41^. Thus, increased 3-HB may have physiological impacts other than as fuel substitutes. In addition, 2-oxoglutarate may also serve as a transcriptional modulator via activation of 2-oxoglutarate oxygenase, which has several biological roles, including demethylation of histones and nucleic acids, and destabilization of transcriptional factor HIF-1^42^. Fasting may modify epigenetic modulation of transcription via such metabolites. Alternatively, these signaling metabolites may promote changes in homeostasis via signaling networks. Interestingly, among nine aging-related metabolites that decrease among the elderly^7^, three (OA, leucine, and isoleucine) greatly increased during fasting, implying that fasting might affect also aging-related compounds. Collectively, fasting appears to provoke a much more metabolically active state than previously realized. Further investigation of fasting metabolomics will provide collective information on physiological responses of many tissues.

## Methods

### Chemicals and reagents

Metabolite standards were purchased from commercial sources (Table S3). Other reagents have been previously described^6,7,43^.

### Ethics statement

Written, informed consent was obtained from all donors in accordance with the Declaration of Helsinki. All experiments were performed in compliance with relevant Japanese laws and institutional guidelines. All protocols were approved by the Ethics Committee on Human Research of Kyoto University Hospital and by the Human Subjects Research Review Committee of the Okinawa Institute of Science and Technology Graduate University (OIST).

### Human experiments

Four healthy, young volunteers (Fig. 1a) fasted for 58 hr. They did not eat or consume any calories, while carrying out their normal routines; however, volunteers imbibed calorie-free drinks. The levels of physical activity and water intake were not monitored. Their blood was sampled on three consecutive weekdays each morning at 9:00 in the laboratory at Kyoto University Hospital (Fig. 1b).

### Blood sample preparation for metabolomic analysis

Human blood sample preparation was performed as described previously^6^. Because some metabolites are extremely labile, our rapid sample preparation insured data reproducibility. From each blood sample three metabolomic samples were prepared: whole blood, plasma, and red blood cells (RBCs). 10 nmol each of HEPES (4-(2-hydroxyethyl)-1-piperazineethanesulfonic acid) and PIPES (piperazine-N, N’-bis(2-ethanesulfonic acid)) were added to each sample to serve as standards.

### LC-MS conditions

Non-targeted, comprehensive LC-MS conditions were as described previously^6,12,44^. Briefly, LC-MS data were obtained using a Paradigm MS4 HPLC system (Michrom Bioresources, Auburn, CA, USA) coupled to an LTQ Orbitrap mass spectrometer (Thermo Fisher Scientific, Waltham, MA, USA). LC separation was performed on a ZIC-pHILIC column (Merck SeQuant, Umeå, Sweden, 150 mm × 2.1 mm, 5 μm particle size). Acetonitrile (A) and 10 mM ammonium carbonate buffer, pH 9.3 (B) were used as the mobile phase, with gradient elution from 80% A to 20% A in 30 min, at a flow rate of 100 μl/min. An electrospray ionization (ESI) source was used for MS detection. Each sample was injected twice (1 μl volume/injection); one with the ESI operated in negative ionization mode and the other in positive ionization mode. Spray voltage and capillary temperature were set to 2.8 kV (negative ESI) or 4.0 kV (positive ESI) and to 350 °C or 300 °C, respectively. Nitrogen was used as the carrier gas. The mass spectrometer was operated in a full scan mode with a 100–1000 *m/z* scan rage and automatic data-dependent MS/MS fragmentation scans.

### LC-MS data processing and analysis

Peak areas of metabolites of interest were measured using MZmine 2 (version 2.21) software^18^ (mzmine.github.io). Data analytical procedures and parameters have been described in Table S4. After peak detection, isotopic peaks were removed and peak lists of individual samples were aligned using their corresponding *m/z* and retention time values. 120 non-selective metabolites were identified for each sample by comparing retention times and *m/z* values of peaks with those of standards (Table S1)^6,12^. In some cases, retention times of isomers overlapped; thus, we designated those peaks by common names (*e.g*., aminobutyrate, pentose phosphate). 2-aminobutyrate, 3-aminobutyrate, and 3-amino(iso)butyrate standards eluted at 11.8, 12.2, and 12.2 min, respectively. Keto(iso)leucine was identified as overlapping peaks of 3-methyl-2-oxopentanoate and 4-methyl-2-oxopentanoate in our detection system. Corresponding peak(s) in metabolomic samples were designated simply as aminobutyrate. Data were exported into spreadsheet format and analyzed with R statistical software (http://www.r-project.org). Statistical analysis was performed using Friedman test. Statistical significance was established at p < 0.05. A post hoc power analysis was conducted with G*Power 3.1.9.2^45^ (http://www.gpower.hhu.de/). The power (1-β error probability) was calculated by statistical test “ANOVA: Repeated measures, within factors” at the significance level (α error probability, p = 0.05). Effect size *f* and correlation among repeated measures were determined from the mean and variance in each group.

## Electronic supplementary material

Supplementary Information

Supplemental Table S1

## Data Availability

Raw LC-MS data in mzML format are accessible via the MetaboLights repository (http://www.ebi.ac.uk/metabolights). Fasting data for the four volunteers are available under accession number MTBLS549.
