# Supplementary material for: Diverse metabolic reactions activated during 58-hr fasting are revealed by non-targeted metabolomic analysis of human blood
Source: Sci Rep. 2019 Jan 29;9:854. doi: 10.1038/s41598-018-36674-9 (PMC6351603; doi:10.1038/s41598-018-36674-9)
Supplement: Supplementary file 1 — Supplementary Information [file 41598_2018_36674_MOESM1_ESM.pdf]

Supplementary information

Diverse metabolic reactions activated during 58-hr fasting are revealed by non-targeted metabolomic analysis of human blood

Takayuki Teruya[1], Romanas Chaleckis[1,3], Junko Takada[1], Mitsuhiro Yanagida[1]* and Hiroshi Kondoh[2]*

[1] G0 Cell Unit, Okinawa Institute of Science and Technology Graduate University (OIST), Okinawa, Japan,

[2] Geriatric unit, Department of Community Network and Collaborative Medicine, Graduate School of Medicine, Kyoto University, Kyoto, Japan

[3] Present address: Gunma University Initiative for Advanced Research (GIAR), Gunma University, Gunma, Japan.

* To whom correspondence should be addressed: Co-corresponding authors.

H. Kondoh, Geriatric unit, Department of Community Network and Collaborative Medicine, Graduate School of Medicine, Kyoto University, Sakyo-ku, Kyoto, 606-8507, Japan

Fax & Tel +81 75 751 3465

Email; hkondoh@kuhp.kyoto-u.ac.jp Lead Contact

M. Yanagida, G0 Cell Unit, Okinawa Institute of Science and Technology Graduate University (OIST), Onna-son, Okinawa, 904-0495, Japan

Tel +81 98 966 8658 Fax +81 98 966 2890

Email; myanagid@gmail.com

Supplemental Table S1. 120 identified blood metabolites. (Excel file)

120 identified metabolites were detected in blood metabolome samples by LC-MS. Compounds were identified using either commercially available standards (STD) or by analysis of MS/MS spectra (MS/MS), if no standard was available. H, M, and L indicate relative abundances of metabolites identified previously[6,7]. ND, not detected. 46 compounds that showed significant ($p<0.05$) increases (>1.5x) or decreases (<0.66x) are highlighted in yellow.

Supplemental Table S2. The post hoc power analysis of the fasting markers.

The probability (p) value was calculated by the Friedman test. Effect size and power were calculated by post hoc analysis using G*Power software (see Method section). NA, not applicable, due to non-significance (p>0.05).

Compounds	Blood			Plasma			RBC		
	p-value	effect size f	power	p-value	effect size f	power	p-value	effect size f	power
2-Hydroxybutyrate	0.0183	1.78	1.00	0.0388	2.08	1.00	0.0388	1.68	1.00
2-Ketobutyrate	0.0183	1.24	1.00	0.0183	1.05	1.00	0.0388	0.66	0.70
3-Hydroxybutyrate	0.0183	2.16	1.00	0.0183	2.14	1.00	0.0388	1.49	1.00
Aminobutyrate	0.0183	2.04	1.00	0.0388	1.09	1.00	0.0183	1.34	1.00
Isoleucine	0.0388	1.05	1.00	0.0388	0.92	0.97	>0.05	NA	NA
Keto(iso)leucine	0.0183	1.65	1.00	0.0183	2.17	1.00	0.0388	1.75	1.00
Ketovaline	0.0183	1.89	1.00	0.0183	2.03	1.00	0.0388	1.49	1.00
Leucine	0.0388	1.21	1.00	0.0388	1.25	1.00	>0.05	NA	NA
Valine	>0.05	NA	NA	0.0388	1.01	0.99	>0.05	NA	NA
Acetyl-carnitine	0.0183	1.09	1.00	0.0183	2.38	1.00	0.0388	0.40	0.27
Decanoyl-carnitine	0.0388	0.64	0.67	0.0183	0.85	0.94	0.0388	1.04	1.00
Dodecanoyl-carnitine	0.0498	0.87	0.90	0.0183	1.08	1.00	0.0183	1.32	1.00
Hexanoyl-carnitine	0.0388	0.58	0.56	0.0388	0.60	0.61	0.0388	0.89	0.96
Isovaleryl-carnitine	>0.05	NA	NA	0.0498	0.67	0.73	0.0388	0.38	0.25
Octanoyl-carnitine	0.0388	1.28	1.00	0.0388	0.83	0.93	0.0498	1.10	1.00
Tetradecanoyl-carnitine	0.0498	0.69	0.76	0.0183	0.85	0.94	0.0183	0.96	0.99
2-Oxoglutarate	0.0183	1.23	1.00	0.0388	0.89	0.96	>0.05	0.59	0.58
cis-Aconitate	>0.05	NA	NA	0.0183	1.24	1.00	>0.05	NA	NA
Citrate	>0.05	NA	NA	0.0388	0.98	0.99	>0.05	NA	NA
Malate	0.0388	0.88	0.96	0.0388	0.66	0.70	0.0498	0.54	0.50
Succinate	>0.05	NA	NA	0.0388	0.99	0.99	>0.05	NA	NA
Nicotinamide	0.0388	0.95	0.98	0.0388	0.58	0.56	0.0388	0.98	0.99
Pantothenate	0.0498	0.42	0.30	0.0388	0.70	0.77	>0.05	NA	NA
Adenine	>0.05	NA	NA	>0.05	NA	NA	0.0183	1.13	1.00
ADP	0.0388	0.73	0.82	>0.05	NA	NA	>0.05	NA	NA
CTP	>0.05	NA	NA	0.0388	0.43	0.32	>0.05	NA	NA
Cytidine	>0.05	NA	NA	0.0388	0.51	0.43	0.0388	1.04	0.98
GTP	>0.05	NA	NA	0.0183	0.75	0.83	>0.05	NA	NA
IMP	>0.05	NA	NA	0.0183	0.62	0.64	>0.05	NA	NA
Urate	0.0388	1.20	1.00	>0.05	NA	NA	0.0388	0.83	0.93
Uridine	0.0388	1.52	1.00	0.0388	1.19	1.00	0.0498	1.20	1.00
Xanthine	0.0388	0.59	0.58	0.0388	0.52	0.46	0.0388	0.56	0.52
6-Phosphogluconate	>0.05	NA	NA	0.0388	0.35	0.21	>0.05	NA	NA
Diphospho-glycerate	0.0388	0.66	0.70	0.0388	1.99	1.00	>0.05	NA	NA
Gluconate	>0.05	NA	NA	0.0388	0.73	0.81	>0.05	NA	NA
Glucose-6-phosphate	>0.05	NA	NA	0.0388	0.83	0.93	>0.05	NA	NA
Glycerol-phosphate	0.0498	0.79	0.89	0.0498	0.68	0.73	>0.05	NA	NA
Pentose-phosphate	>0.05	NA	NA	0.0498	0.83	0.92	>0.05	NA	NA
Sedoheptulose-7-phosphate	>0.05	NA	NA	0.0388	0.75	0.85	>0.05	NA	NA
Ergothioneine	0.0498	0.18	0.09	0.0183	0.25	0.13	0.0388	0.20	0.10
Ophthalmic acid	0.0183	3.31	1.00	not detected			0.0183	1.81	1.00
Carnosine	>0.05	NA	NA	not detected			0.0183	0.44	0.33
Aspartate	>0.05	NA	NA	0.0388	0.92	0.98	>0.05	NA	NA
Dimethyl-arginine	>0.05	NA	NA	0.0388	0.68	0.73	0.0388	0.87	0.95
Lysine	0.0388	0.84	0.93	0.0388	0.67	0.72	>0.05	NA	NA
N-Acetyl-(iso)leucine	>0.05	NA	NA	0.0388	0.87	0.95	0.0388	0.88	0.96

Supplemental Table S3. Standards for peak identification.

Previously reported standards and their vendors are found in Pluskal et al.[12] or Chaleckis et al.[6,7]

Compound	Vendor	Product name	Catalog #
2-Hydroxybutyrate	Sigma-Aldrich	2-Hydroxybutyric acid sodium salt	220116-5G
2-Ketobutyrate	Sigma-Aldrich	2-Ketobutyric acid	K401-5G
3-Hydroxybutyrate	Sigma-Aldrich	3-Hydroxybutyric acid	166898-1G
Aminobutyrate	Wako	DL-2-Aminobutyric acid	015-14542
	Wako	DL-3-Aminobutyric acid	019-02411
	TCI	DL-3-Aminoisobutyric Acid Hydrate	A0324
Keto(iso)leucine	Sigma-Aldrich	(±)-3-Methyl-2-oxovaleric acid sodium salt	K7125-5G
	Sigma-Aldrich	4-Methyl-2-oxovaleric acid	68255-1G
Ketovaline	Combi-Blocks	3-Methyl-2-oxo-butanoic acid	ST-4537

Supplemental Table S4. Methods and parameters used for data processing with MZmine 2.21.

**1. Raw data methods / Raw data file import**	
Filename	choose all data files
**2. Raw data methods / Peak detection / Mass detection**	
Mass detection, mass detector	Exact mass
Noise level (positive ionization mode)	5E3
Noise level (negative ionization mode)	1E3
Mass detection, MS level	1
**3. Raw data methods / Peak detection / FTMS shoulder peaks filter**	
Mass resolution	60,000
Peak model function	Lorentzian extended
**4. Raw data methods / Peak detection / Chromatogram builder**	
Min time span (min)	0.1
Min height	1E4
m/z tolerance	0.003 m/z or 10 ppm
**5. Peak list methods / Peak detection / Smoothing**	
Filter width	5
**6. Peak list methods / Peak detection / Chromatogram deconvolution**	
Algorithm	Local minimum search
Chromatographic threshold	70%
Search minimum in RT range (min)	0.3
Minimum relative height	1%
Minimum absolute height	1E4
Min ratio of peak top/edge	1.5
Peak duration range (min)	0-10
**7. Peak list methods / Isotopes / Isotope peak grouper**	
m/z tolerance	0.02 m/z or 20 ppm
Retention time tolerance	0.1 min
Maximum charge	2
Representative isotope	Most intense
**8. Peak list methods / Alignment / Join aligner (run separately for negative ionization mode and positive ionization mode data)**	
m/z tolerance	0.003 m/z or 10 ppm
Weight for m/z	10
Retention time tolerance	0.5 min
Weight for RT	10
**9. Peak list methods / Gap filling / Same RT and m/z range filling**	
m/z tolerance	0.003 m/z or 10 ppm
**10. Peak list methods / Identification / Custom databese search**	
(custom CSV database of previously identified compounds was used)	

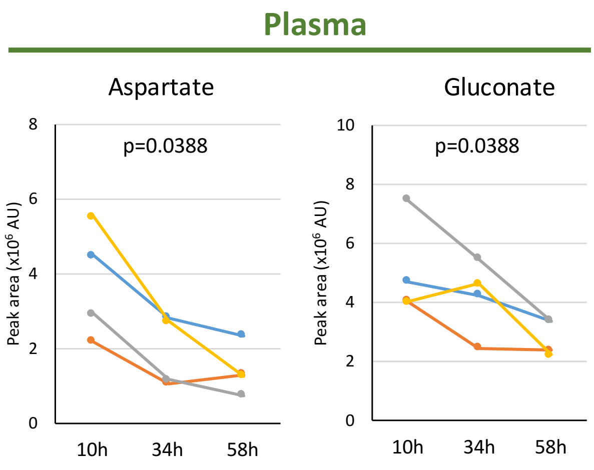

Supplemental Figure S1. Aspartate and gluconate decreased <0.66x in plasma during fasting. Changes of aspartate and gluconate in peak area were observed in plasma samples of four volunteers. P-values are presented to show the significance of serial change until 58 hr by Friedman test.

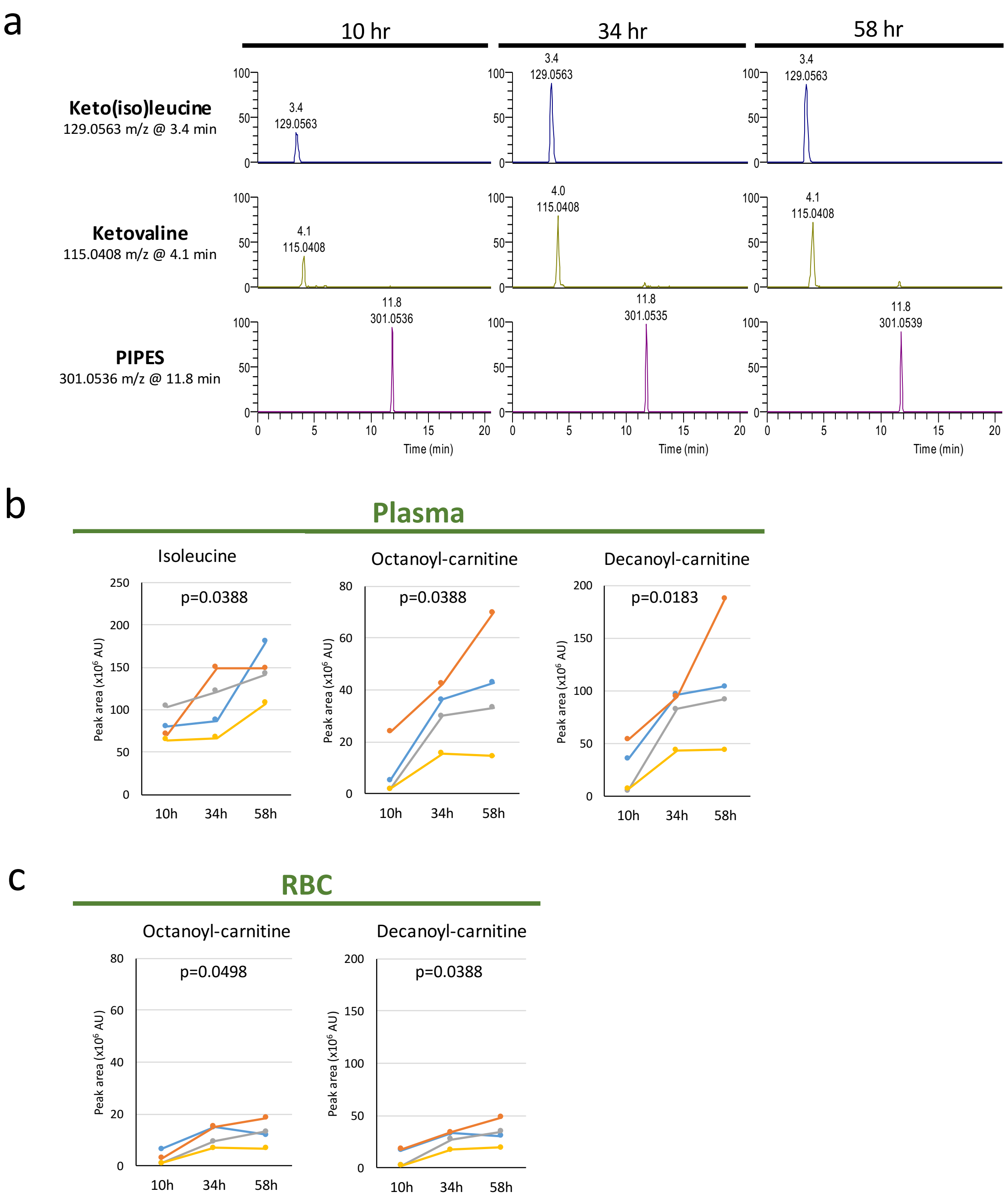

Supplemental Figure S2. Increases of BCAAs and carnitines during fasting.

(**a**) Peaks of keto(iso)leucine and ketovaline increased significantly during 58 hr of fasting. PIPES was used as an internal control. (**b, c**) Concentration changes of previously known fasting metabolite markers, isoleucine, valine, octanoyl-, and decanoyl-carnitine, as determined by LC-MS. Profiles in plasma (**b**) and in RBCs (**c**) are shown. In each panel, p-values were obtained by Friedman test.

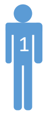

Volunteer 1

Compounds	change 34 h/10 h	Compounds	change 58 h/10 h
3-Hydroxybutyrate	25.40	3-Hydroxybutyrate	65.56
Octanoyl-carnitine	11.66	2-Ketobutyrate	16.03
2-Ketobutyrate	10.07	2-Hydroxybutyrate	11.85
2-Hydroxybutyrate	6.56	Octanoyl-carnitine	8.74
Hypoxanthine	3.69	Adenine	7.32
Adenine	3.42	Ophthalmic acid	4.45
Ketovaline	2.93	Dodecanoyl-carnitine	3.67
Glyceric acid	2.81	Keto(iso)leucine	3.57
Dodecanoyl-carnitine	2.67	Ketovaline	3.46
Uridine	2.57	Glyceric acid	3.45
Keto(iso)leucine	2.57	Hypoxanthine	3.43
Glyceraldehyde-3-phosphate	2.56	Aminobutyrate	3.37
Ophthalmic acid	2.53	Glyceraldehyde-3-phosphate	3.37
Nicotinamide	2.51	Uridine	3.30
Xanthine	2.48	Tetradecanoyl-carnitine	3.12

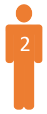

Volunteer 2

3-Hydroxybutyrate	24.75	3-Hydroxybutyrate	25.08
2-Hydroxybutyrate	11.74	2-Ketobutyrate	12.71
2-Ketobutyrate	9.42	2-Hydroxybutyrate	12.50
Tetradecanoyl-carnitine	4.12	Aminobutyrate	5.54
Leucine	3.51	N-Acetyl-glutamate	5.51
Ketovaline	3.15	Ophthalmic acid	3.68
Valine	3.04	Ketovaline	3.55
Isoleucine	2.90	Tetradecanoyl-carnitine	3.55
N-Acetyl-glutamate	2.90	2-Oxoglutarate	3.26
Dodecanoyl-carnitine	2.89	Octanoyl-carnitine	3.20
Xanthine	2.80	Keto(iso)leucine	2.98
2-Oxoglutarate	2.67	Dodecanoyl-carnitine	2.83
Keto(iso)leucine	2.67	Leucine	2.71
Octanoyl-carnitine	2.65	Xanthine	2.67
Ophthalmic acid	2.54	Valine	2.58

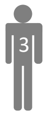

Volunteer 3

3-Hydroxybutyrate	20.02	3-Hydroxybutyrate	73.10
Decanoyl-carnitine	16.92	Decanoyl-carnitine	9.68
Tetradecanoyl-carnitine	13.18	2-Hydroxybutyrate	8.79
Octanoyl-carnitine	12.42	Octanoyl-carnitine	8.13
Dodecanoyl-carnitine	10.86	Tetradecanoyl-carnitine	8.03
2-Hydroxybutyrate	4.90	Aminobutyrate	7.63
Xanthine	4.75	Dodecanoyl-carnitine	6.77
Uridine	3.53	2-Ketobutyrate	6.39
Hexanoyl-carnitine	2.84	Xanthine	5.35
2-Ketobutyrate	2.72	Uridine	3.65
Keto(iso)leucine	2.44	Adenine	3.48
Ketovaline	2.37	Ketovaline	2.66
Adenine	2.35	Keto(iso)leucine	2.60
Hypoxanthine	2.28	Ophthalmic acid	2.43
Cytidine	2.05	Hexanoyl-carnitine	1.92

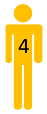

Volunteer 4

3-Hydroxybutyrate	9.45	3-Hydroxybutyrate	68.06
Tetradecanoyl-carnitine	8.65	2-Hydroxybutyrate	22.21
Decanoyl-carnitine	6.12	2-Ketobutyrate	15.63
Octanoyl-carnitine	5.46	Tetradecanoyl-carnitine	12.27
2-Hydroxybutyrate	5.15	Decanoyl-carnitine	5.75
2-Ketobutyrate	4.73	Dodecanoyl-carnitine	5.65
Dodecanoyl-carnitine	4.11	Xanthine	5.13
Xanthine	3.93	Octanoyl-carnitine	4.39
Uridine	2.46	Keto(iso)leucine	4.36
Keto(iso)leucine	2.15	Ketovaline	4.03
Hexanoyl-carnitine	2.12	Ophthalmic acid	3.67
Ketovaline	2.06	Aminobutyrate	3.37
Ophthalmic acid	1.75	Uridine	2.99
Urate	1.58	Acetyl-carnitine	2.36
Aminobutyrate	1.57	Isoleucine	2.12

Supplemental Figure S3. Individual variation in fasting responses.

Fifteen metabolites that changed most significantly in four volunteers are listed, arranged in order of their degree of change. Butyrates are shown in red, while acylcarnitines and BCAAs are in green and blue, respectively. Left panels are comparison of levels between 10 and 34 hr in each volunteer, while comparison of those between 10 and 58 hr of fasting are shown in right panels.

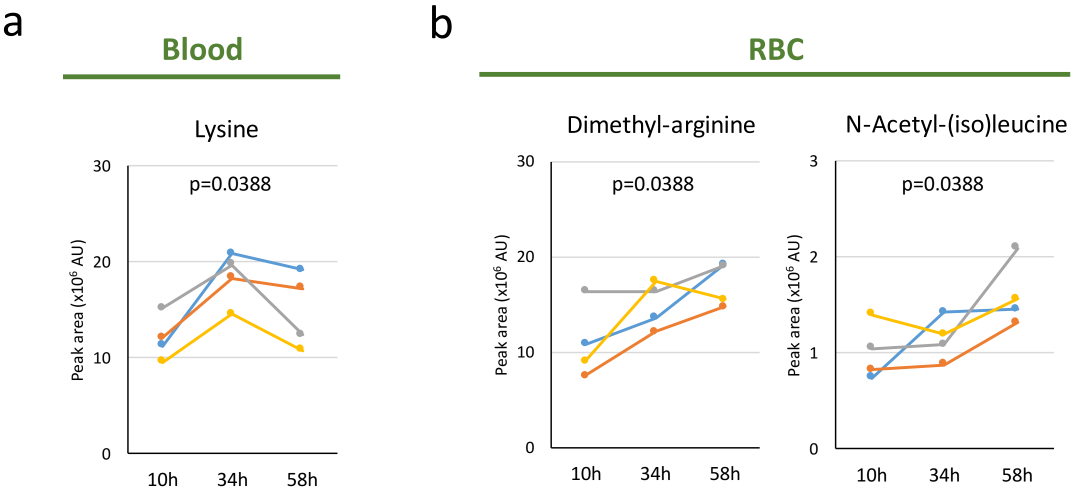

Supplemental Figure S4. Lysine concentration increased in whole blood and two amino acid derivatives increased in RBCs during fasting.

(**a**) Lysine displayed significant changes in whole blood. (**b**) Dimethyl-arginine and N-acetyl-(iso)leucine increased during fasting in RBCs. In each panel, p-values were obtained by Friedman test.
